# Oncolytic virotherapy as an immunotherapeutic strategy for multiple myeloma

**DOI:** 10.1038/s41408-017-0020-0

**Published:** 2017-12-05

**Authors:** Daniel E. Meyers, Satbir Thakur, Chandini M. Thirukkumaran, Don G. Morris

**Affiliations:** 1 Department of Oncology, University of Calgary, Calgary, AB Canada; 2 Tom Baker Cancer Centre, Calgary, AB Canada

## Abstract

Multiple Myeloma (MM), a clonal malignancy of antibody-producing plasma cells, is the second most common hematologic malignancy and results in significant patient morbidity and mortality. The high degree of immune dysregulation in MM, including T cell imbalances and up-regulation of immunosuppressive checkpoint proteins and myeloid derived suppressor cells, allows this malignancy to escape from host immune control. Despite advances in the therapeutic landscape of MM over the last decade, including the introduction of immunomodulatory drugs, the prognosis for this disease is poor, with less than 50% of patients surviving 5 years. Thus, novel treatment strategies are required. Oncolytic viruses (OV) are a promising new class of therapeutics that rely on tumour specific oncolysis and the generation of a potent adaptive anti-tumour immune response for efficacy. To date, a number of OV have shown efficacy in pre-clinical studies of MM with three reaching early phase clinical trials. OVs represent a rational therapeutic strategy for MM based on (1) their tumour tropism, (2) their ability to potentiate anti-tumour immunity and (3) their ability to be rationally combined with other immunotherapeutic agents to achieve a more robust clinical response.

## Introduction

Multiple Myeloma (MM), a clonal malignancy of antibody-producing plasma cells, is the second most common hematologic malignancy and was responsible for an estimated 13 000 fatalities in Americans during 2016^1^. Active MM manifests clinically with hypercalcemia, renal failure, anemia, osteolytic lesions and recurrent bacterial infections—all resulting from either the hyperproliferation of malignant plasma cells, or sequelae of the accumulating dysfunctional monoclonal immunoglobulin protein that they produce. The past decade has seen significant changes in the landscape of MM treatment, including the advent of novel agents such as thalidomide derivatives (lenalidomide, pomalidomide) and proteasome inhibitors (bortezomib, carfilzomib) for use in both transplant- and non-transplant eligible patients^2^. Despite the significant improvement in the prognosis of MM during this time frame overall survival rates are still modest, with less than 50% of patients surviving 5 years^3^. Thus, novel treatment strategies are clearly needed.

It has been more than 100 years since the discovery that viruses can play a role in the treatment of cancer^4^. Over the course of the 20^th^ century, further anecdotal evidence emerged that viral infection could induce remission in various cancer types^5, 6^, including MM^7^. It is now well-understood that a wide range of viruses have the ability to specifically infect and kill cancer cells. Despite variable interest in the use of oncolytic viruses (OV) as an immunotherapy over the past century, there has been a recent resurgence in the field. In 2015, the FDA approved the first OV for clinical use—an oncolytic herpes simplex virus for use in metastatic melanoma^8^. As experience with OV therapy accumulates, MM has begun to emerge as one prime candidate for its use.

## OV basics

The basis of OV therapy is that certain viruses can selectively infect and lyse cancer cells, while leaving non-malignant cells unaffected. The clinical applicability of OV utilizes the biology that underscores typical host-virus interactions; ideally, the OV activates the innate and adaptive immune responses generated in response to viral infection, but re-directs them specifically towards the tumour. Initial oncolysis unmasks tumour neo-antigens that may have otherwise been functionally hidden from the host’s immune system. Thus, success of OVs as a cancer therapeutic relies on both tumour oncolysis and the subsequent activation of an anti-tumour immune response. However, the same immune response that promotes activity against malignant cells, can also decrease the effectiveness of OV, as the neutralization of the viruses by the host may hinder their ability to replicate through the tumour. This delicate interplay between the anti-tumour and anti-viral effects of the immune system ultimately dictates the potential effectiveness of OV as cancer therapeutics.

Viral proteins and nucleic acids are differentiated from host cellular components by pattern recognition receptors, called toll-like receptors (TLRs). Binding of these viral structures to TLRs leads to the expression of inflammatory cytokines like interferon (IFN) and tumour necrosis factor, ultimately leading to the up-regulation of the hosts antiviral machinery, including double stranded RNA protein kinase R (PKR). Interestingly, the PKR pathway may be abnormal in cancer cells and as such, viral clearance from these cells may be attenuated^9^. Tumour cell death following viral oncolysis activates the non-specific, innate immune system. Ultimately, the local release of the inflammatory cytokines leads to the maturation of antigen-presenting cells, including dendritic cells (DCs). DCs bring the tumour antigens to peripheral lymphoid tissue, where they activate antigen- naïve CD4^+^ and CD8^+^ T cell responses. This arms the hosts immune system with the ability to locate and destroy malignant cells that were previously “hidden” from the host immune system.

Priming and activation of the adaptive immune system is known to play a major role in the antitumor effects of OV therapy. As an example, the therapeutic effect of oncolytic reovirus, in combination with sunitinib, was completely lost in a murine model of renal cell carcinoma (RCC) secondary to CD8^+^ T cell depletion^10^. However, if the CD8^+^ population was maintained, cured mice were protected from subsequent tumour re-challenge. Similar findings have been demonstrated in other OV genera and tumour histologies^11, 12^. It is also important to distinguish the anti-tumour response induced by the adaptive immune system and that mediated by direct tumour lysis. For example, B16ova melanoma cells are resistant to direct reovirus (RV) oncolysis, owing to their low expression of RV receptor junctional adhesion molecule-A (JAM-A). However, in a study by Prestwich and colleagues^13^ immunocompetent mice inoculated with these melanoma tumours and treated with with RV-loaded T cells had significant reduction in splenic and lymph node metastases, as well as an up-regulated IFN production of splenocytes secondary to tumour cell exposure, suggestive of an active adaptive anti-tumour response even in the absence of direct tumour lysis. As will be discussed, both direct tumour oncolysis and an adaptive anti-tumour response are critical components to the success of OV therapy in MM.

## Immune dysregulation in multiple myeloma

The capacity for the host immune system to recognize and destroy MM cells remains a critical challenge in the treatment of this malignancy. The fundamental importance of immune recognition of MM cells is highlighted by the therapeutic efficacy of allogenic hematopoietic stem cell transplantation (HSCT) in these patients^14, 15^. Underscoring at least part of HSCT efficacy is the graft-vs.-myeloma effect, demonstrated by the sustained disease remissions obtained with donor lymphocyte infusion following disease relapse post-HSCT^16^.

However, the high degree of immune dysregulation in MM allows it to progress while largely evading host immune surveillance, despite potential immune recognition. Thus, utilizing therapeutic approaches that augment the immunogenicity of MM have the potential to offer additional clinical benefit. OVs are one such example.

### Immune checkpoints: PD-1/PD-L1

MM cells express a number of different molecules that abrogate the ability of the host immune system to respond against them^17^. Immune checkpoint proteins, such as those involving the programmed death (PD)-1/PD-L1 axis are critical in the maintenance of self-tolerance, but are often overexpressed by malignant cells in order to evade detection and subsequent destruction by the host immune system^18^. Further, PD-L1 overexpression has been tied to a worsened prognosis in many of these cases^19, 20^.

Multiple groups have reported overexpression of PD-L1 on malignant plasma cells of MM^21, 22^, but not on plasma cells from healthy controls^23^. Interestingly, PD-L1 expression has been suggested to increase as MM undergoes its natural progression from monoclonal gammopathy of unknown significance to active MM^24^. Furthermore, it has been suggested that activity of the PD-1/PD-L1 axis in MM is associated with a more aggressive clinical disease^25^. Additionally, immune checkpoint proteins of the PD-1/PD-L1 axis have also been noted to be overexpressed on immune populations within the MM microenvironment, such as DCs^22, 26^, myeloid derived suppressor cells (MDSCs)^27^, and regulatory T cells (Tregs)^28^.

Disrupting this immunosuppressive axis with monoclonal antibodies has shown preclinical promise to date. Specifically, PD-1 blockade was found to restore the potential for DCs to evoke CD8^+^ T-cell killing of myeloma targets in vitro^21^. Additionally, PD-L1 blockade has been shown to enhance T-cell response to autologous DC’s and induce CD8^+^ T-cells, leading to marked cytolytic activity against MM cells^22^. Disruption of this axis has also shown promise in murine models, with extended survival seen among treated groups compared to respective controls^29, 30^. These findings have transitioned to investigations in humans, where a number of clinical trials involving PD-1/PD-L1 blockade in MM are currently ongoing^31^.

### T cell imbalance

It is recognized that patients with MM have both quantitative and functional T-cell deficits, but with the exception of high rates of herpes zoster and related viral infections^32^, MM patients do not seem do display typical signs of clinical T-cell immunodeficiency. Thus, there seems to be spared T-cell immunity against most external antigens, but impaired immunity against the myeloma itself. Historical data has suggested that a decreased CD4^+^/CD8^+^ ratio exists in MM^33, 34^, and that this ratio decreases further as the disease progresses^35^. In contrast, more recently, Zelle-Rieser and colleagues found a trend to decreased CD4^+^/CD8^+^ ratio in treated as compared to untreated MM patients^36^. However, this same group also found that T cells within the MM tumour microenvironment displayed specific cellular markers of exhaustion (PD-1, CTLA-4, 2B3,CD160) and senescence(CD57,CD28^−^)^37^.

Among CD4^+^ T-cell subsets, the balance between pro-inflammatory T helper 17 (Th17) cells and immunosuppressive Tregs is known to be a major factor in immune control of malignancy^38, 39^. Specifically, patients with MM generally have an increased number of Tregs compared to healthy controls^40–43^, but this notion is controversial^44, 45^. Differences in subset quantification likely can be attributed to technical differences in assay methodology and selection of patients^46^. Nonetheless, the relationship between Th17/Tregs appears to hold some clinical significance. For example, a study by Bryant and colleagues^47^ found that there is a significantly higher Th17/Treg ratio in long term survivors (>10 years from diagnosis) of MM compared with patients with less than 10 years of follow-up. Additionally, an increase in Treg levels was found to be predictive of both poorer overall survival and shorter progression-free survival in patients undergoing HSCT for treatment of MM^48^.

### Myeloid derived suppresor cells (MDSCs)

Healthy individuals possess a small number of immature myeloid cells in their bone marrow that typically differentiate into functioning DCs, granulocytes and macrophages. However, under the stimulus of specific cytokines such as granulocyte macrophage colony stimulating factor and IL-6, or from signaling proteins like vascular endothelial growth factor, differentiation can be impeded and instead these cells rapidly develop into immature MDSCs. MDSCs are defined in humans as CD11b^+^CD33^+^HLA-DR^low^/^-^ cells. Human MDSCs have classically been divided into two unique subsets: a monocytic type, identified as CD14^+^, and a granulocytic type identified as CD15^+^^49, 50^. These cells have the capacity to inhibit T cell function by producing arginase-1, reactive oxygen species and nitric oxide^51, 52^. There are a number of other mechanisms through which MDSCs can be immunosuppressive, including suppressing NK activity^53^, and decreasing migratory capacity of naïve T cells through the down-regulation of L-selectine^54^. Patients with MM are known to have an increased number of MDSCs^55, 56^, but there is controversy over their link to clinical outcome^47, 57^.

MDSCs represent an important clinical target in MM, not only for their role in the immunosuppressive tumour microenvironment, but also for their capacity to differentiate into osteoclasts^58^, which are responsible for a major source of patient morbidity. One such approach at targeting MDSC in MM has come through the use of phosphodiesterase-5 (PDE) inhibitors, such as tadalafil, to decrease their suppressor function. By inhibiting MDSC-dependent nitric oxide and arginase-1, PDE5 inhibitors have been shown to increase tumour infiltration by cytotoxic T-cells and improve the anti-tumour efficacy of adoptive T cell therapy in relevant mouse models^59^. Unfortunately, a phase II clinical trial evaluating this mechanism of MDSC inhibition in MM was suspended due to lack of efficacy, likely underscored by the absence of detectable MDSC counts in any of the patients at baseline^60^. Overall, despite being an important regulator of the immunosuppressive milieu surrounding MM the pre-clinical success of targeting MDSC and future translational opportunities are not yet clear^61^. One future avenue of investigation may be in the multi-tyrosine kinase inhibitor sunitinib, as it has been shown to decrease intratumoral and splenic MDSC numbers in a murine model of RCC^10^.

## Evidence for oncolytic virotherapy in multiple myeloma

OVs have been exploited for their anti-tumour effects across a myriad of solid and hematologic malignancies^62^. In MM specifically, recent pre-clinical studies utilizing OV have focused on three RNA viruses (measles virus, RV, vesicular stomatitis virus) and two DNA viruses (myxoma virus, vaccinia virus). (Table 1) Pre-clinical success to date has led to early-phase clinical trials to evaluate the efficacy of measles virus (MV), RV and vesicular stomatitis virus (VSV) in MM.

**Table 1:** OVs with recent pre-clinical success in MM and their characteristics

	**MV**	**MYXV**	**RV**	**VSV**	**VV**
Family	Paramyxoviridae	Poxviridae	Reoviridae	Rhabdoviridae	Poxviridae
Genome Type	ss(-)RNA	dsDNA	dsRNA	ss(-)RNA	dsDNA
Genome Size	~ 15 kb	~ 160 kb	~ 24 kb	~ 11 kb	~ 190 kb
Virion	Enveloped	Enveloped	Naked	Enveloped	Enveloped
Cell Receptors	SLAM & CD46	?	JAM-1	LDLR	?
Genetically Modifiable	Y/Easy	Y/Easy	Y/Difficult	Y/Moderate	Y/Easy
Achievable Titre (PFU/mL)	>10^9^	>10^9^	>10^9^	>10^9^	>10^9^
Pre-Clinical*	Y	Y	Y	Y	Y
*In vitro*	[64,65]	[78,80–81]	[90–92]	[110]	[100,102]
*In vivo*	[64,65,67]	[82]	[91,97,116]	[110,111]	[102,104]
Clinical	Y	N	Y	Y	N

### Measles virus (MV)

MV is a negative-stranded RNA virus belonging to the viral family *Paramyxoviridae*, and is the pathogen responsible for the infectious measles syndrome. MV has a ~ 15 kb genome containing 6 genes and encoding 8 proteins: Fusion, hemagglutinin, large, matrix, nucleocapsid, phospho, and two accessory proteins—C and V. MV enters cells through interactions of the hemagglutinin protein with the signaling lymphocytic activation molecule (SLAM) receptor or CD46. After cell entry, MV mediates its cytopathic effects via the expression of the hemagglutinin and fusion proteins on the infected cell surface ultimately leading to characteristic syncytial formation^63^.

Due to the increased expression of CD46 on MM cells^64^, it has been demonstrated to be susceptible to MV-mediated oncolysis^65, 66^. The existence of anti-MV antibodies in the large proportion of patients who have been vaccinated against this virus potentially negates its oncolytic potential. This problem has been overcome in pre-clinical models of MM through a number of strategies, including the use of activated T cell carriers^66, 67^ and lethally irradiated MM (MM1) cell carriers^68^. One modified MV encoding the human thyroidal sodium-iodide symporter (MV-NIS) demonstrated exciting therapeutic potential in MM. The NIS construct allows for the concentration of radioiodine, which can be utilized both for imaging with a γ camera, PET and SPECT/CT, or in therapeutic synergy with β-emitting radioiodine^65, 69, 70^. The study by Dingli and colleagues^65^ demonstrated that MV-NIS could induce significant tumour regression in KAS6/1 myeloma xenografts, as well as in MV-resistant MM1 tumours when combined with I^131^ radiotherapy.

The pre-clinical success of oncolytic MV, and specifically MV-NIS has led to two early-phase clinical trials in humans (NCT00450814, NCT02192775) in combination with the immune-modulatory drug cyclophosphamide (Table 2). The recently published results from NCT00450814 demonstrated that MV-NIS is capable of productively replicating before being cleared by the host immune system, and has a reasonable safety profile. Further, one patient treated had a complete remission that lasted for over 9 months, while others had variable and transient drops in their serum free light chains^71^. UARK 2014-21 (NCT02192775), a phase II trial being conducted at the University of Arkansas, is also evaluating MV-NIS with cyclophosphamide in MM. This trial will involve administering a single dose of intravenous MV-NIS followed by a 4 day course of cyclophosphamide. There are currently no data available from this trial.

**Table 2:** Clinical trials of OVs in MM to date

**Virus**	**Name**	**Mods**	**Phase**	**Combination**	**Status**	**Citation**
Measles	MV-NIS	NIS addition	I/II	+/- Cyclophosphamide	Active	^71^
			II	Cyclophosphamide	Active	NCT02192775
Reovirus	Reolysin	-	I	Lenalidomide or Pomalidomide	Active	NCT03015922
				Bortezomib + Dexamethasone	Active	NCT02514382
				-	Complete	^93^
				Carfilzomib + Dexamethasone	Suspended	NCT02101944
Vesicular stomatitis	VSV-IFNβ-NIS	NIS + IFNβ addition	I	-	Active	NCT03017820

### Myxoma virus (MYXV)

Myxoma virus (MYXV), a large double-stranded DNA virus belonging to the *Poxviridae* family, is a relatively new OV agent. Unlike other poxviruses which can infect a wide variety of mammalian hosts, MYXV exhibits a high degree of species-specific tropism to lagomorphs, of which rabbits are the most notable member. Other than lagomorphs, in which infection causes the lethal disease myxomatosis, MYXV cannot infect any other vertebrate species, including humans^72^. Most poxviruses, including MYXV, do not display cellular tropism based on capacity for cell receptor binding. Rather, the inability of this virus to infect, replicate in, or kill the majority of non-malignant cells can be explained by the interplay between the virus and a number of host signaling pathways. For example, pathways that lead to the up-regulation of the host’s innate inflammatory response molecules restrict MYXV replication, including type I interferon (IFN)^73^ and tumour necrosis factor^74^. It is well known that human cancers have deficient IFN responses and thus unsurprisingly, 70% of human cancer cell lines tested from the National Cancer Institute were found to be susceptible to MYXV oncolysis^75^. The mechanisms through which MYXV has cancer tropism are incompletely understood. However, one key relationship that has emerged is between the MYXV-encoded ankyrin-repeat host range factor, M-T5, and Akt in cancer cells. Interaction between the two leads to enhanced phosphorylation of Akt^76^. Additionally, inhibiting Akt can reduce MYXV tropism^77^, thus highlighting the link between MYXV cancer cell tropism and signal transduction pathways typically over-activated in cancer. Due to its inability to infect non-malignant human host cells, MYXV would theoretically have an excellent safety profile as an OV, and would not face the challenge of pre-existing host antibodies.

Bartee and colleagues^78^ demonstrated that MYXV could infect and induce apoptosis in all human MM cell lines tested in vitro. Additionally, as has been shown previously with RV^79^, they found that MYXV could inhibit systemic in vivo engraftment of human MM cells into immunodeficient mice, by effectively purging primary CD138^+^ MM cells contaminating patient hematopoietic products^78^. Interestingly, MYXV ability to induce apoptosis in MM cells was independent of viral replication, as infected MM cells died before the virus could complete its replication cycle^78^. Thus, understanding the mechanisms of MYXV induced cell death would help facilitate strategies to increase its overall efficacy. Recently, Bartee and colleagues^80^ found that MYXV infection led to MM cell apoptosis through the activation of the extrinsic initiator caspase-8. They postulated that activation of caspase-8 was independent of extrinsic death ligands and instead correlated with depletion of cellular inhibitors of apoptosis, likely mediated by viral-mediated host protein shutoff^80^. Further support for using MYXV as a purging agent for HSCT was illustrated by Villa and colleagues^81^. They found that MYXV can bind to and enter activated T cells, but that infection attenuates their proliferation and secretion of inflammatory cytokines thus decreasing the risk of graft-vs.-host-disease^81^. Furthermore, infected T cells could effectively deliver live OV to residual MM cells, thus augmenting the graft-vs.-myeloma effect.

MYXV has also recently been shown to have potential as an OV in vivo^82^. In mice bearing disseminated MM, administration of intravenous MYXV led to elimination of 70–90% of malignant cells within 24 h, while maintaining the integrity of the hematopoietic bone marrow niche^82^. As is the goal of OV therapy in general, MYXV in this setting also induced a potent anti-MM CD8^+^ T cell response which localized to the bone marrow and completely eradicated established MM in some animals. Although no clinical trials have currently been initiated utilizing oncolytic MYXV in MM, the pre-clinical results are certainly promising.

### Reovirus (RV)

RV is a double-stranded RNA virus belonging to the *Reoviridae* family. This non-enveloped RNA virus proliferates within the cytoplasm of infected cells, and typically begins transcription by producing viral RNAs that help both in replication and also activation of the host dsRNA-activated protein kinase (PKR) pathway. Its demonstrated success as an OV can likely be explained by its fulfillment of many key attributes required of an effective OV: it exploits common signaling pathways utilized by malignant cells, is genomically stable, is easily manufactured and has minimal human toxicity. Reovirus is commonly isolated from both human enteric and respiratory tracts, but is minimally pathogenic^83^. RV tropism to malignant cells is underscored by the aberrant intracellular signaling pathways found in transformed cells, particularly the *Ras* pathway^84^. The mechanism underpinning the link between RV infection and the *Ras* pathway lies in protein kinase R (PKR). Typically, PKR is phosphorylated in response to dsRNA infection, which leads leads to a downstream phosphorylation of eukaryotic translational intrinsic factor, ultimately leading to the termination of viral gene translation^85^. However, in cells that have *Ras* activation, PKR phosphorylation is down-regulated, which leads to the propagation of viral replication^84^. However, *Ras* activity is insufficient to explain RV tropism to malignant cells as it has been shown *Ras* activity does not always correlate with susceptibility to infection^86, 87^. It has been suggested that JAM-A, an immunoglobulin superfamily protein expressed on a variety of hematopoietic cells^88^, is implicated in successful RV infection and oncolysis^89^. In MM, specifically, JAM-A and not *Ras* has been demonstrated to be necessary for RV infection^90^.

Although the mechanisms are not fully elucidated, MM has been shown to be susceptible to RV infection and oncolysis in pre-clinical studies^79, 90, 91^. It has demonstrated that both MM cell lines and patient specimens can be effectively killed with RV in vitro through the induction of apoptosis^91^ and autophagy^92^. Notably, the oncolytic effects in MM were tumour specific as RV-exposed HSCs were able to re-constitute a normal hematopoietic compartment in a mouse model^91^. By using a mouse model that partially recapitulates human MM, it has also been demonstrated that RV can serve as a successful ex vivo purging agent for HSCT^79^. In this study, mice treated with live RV-purged autografts exhibited 100% survival in comparison to mice receiving dead virus-purged controls^79^. Furthermore, recent in vivo work from our group in the Vk*MYC murine model demonstrates that adding the proteasome inhibitor bortezomib to RV potentiates successful oncolysis by increasing viral replication, and enhances the anti-tumour adaptive immune response leading to significant increases in surivival. (Unpublished data) The success of these pre-clinical studies has been leveraged into a number of early phase clinical trials.

A phase I trial of single agent RV (Reolysin) in patients with relapsed MM was completed with results reported in 2014^93^. This study demonstrated that Reolysin treatment was well tolerated and associated with modest RV myeloma cell entry, but negligible intracellular RV protein production. As such, RV does not seem to be effective in MM as a monotherapy, which is consistent with other clinical data obtained from solid tumour sites^94^. Interestingly, correlative studies from the Phase I trial demonstrated that patients’ MM cells did not display significant JAM-1 expression, consistent with RV resistance and the necessity of sufficient viral entry to mediate tumour cell killing^90, 95^. There are two more active clinical trials currently utilizing RV in MM; NCT03015922, in which Reolysin is being tested in combination with the immunomodulatory drug lenalidomide or pomalidomide, and NCT02514382, utilizing Reolysin with bortezomib and dexamethasone. Interim results have demonstrated that while safe, treatment results in stable disease in only half of evaluable patients (n = 3)^96^. One strategy to improve RV as a therapeutic agent for MM would be to combine it with a histone deacetylase inhibitor(HDAC-i). Stiff and colleagues^97^ recently demonstrated that in vitro exposure of MM cells to HDAC-I increased their expression of JAM-1, and dual treatment with RV led to the potentiation of MM cell killing both in vitro and in murine models in vivo.

### Vaccinia virus (VV)

Like MYXV, vaccinia virus (VV) is a member of the *Poxviridae* family and has a large ~190 kb dsDNA genome. Due to its high degree of immunogenicity, eliciting strong cellular and humoral immune responses^98^, VV has been utilized as a vaccine that has been essential in the eradication of smallpox. VV propensity to infect and replicate in malignant cells is multi-mechanistic, utilizing cellular epidermal growth factor receptor/Ras signaling, cellular thymidine kinase (TK) levels, and resistance to type I IFN^99^. The relative ease with which the genome of VV can be manipulated and its replication within the cytosol exclusively are two additional features which make it an ideal candidate as an OV.

The first in vitro study investigating oncolytic VV in MM was conducted by Deng and colleagues^100^. Utilizing a VV with a dual TK and vaccinia growth factor deletion and green fluorescent protein insertion (vvDDGFP)^101^, this group demonstrated that MM cell lines and ex vivo patient tumours had reduced viability when exposed to vvDDGFP. Additionally, a subcutaneous xenograft murine model of MM demonstrated significant reduction in tumour burden and consequent survival advantage over controls^100^. Other modified VV have also shown anti-tumour activity against MM^102, 103^. Recently, two novel oncolytic VV (TK deletion) that express anti-tumour genes miR-34a and Smac were developed for pre-clinical testing in MM^102^. The results demonstrated that these VV could effectively infect MM cell lines and enhance exogenous gene expression. Moreover, the combination of these OVs in vitro and in vivo synergistically inhibited tumour growth. The mechanism underpinning the findings was proposed to be via miR-34a induced Bcl-2 blockade and amplification of resultant apoptosis by Smac^102^. Another group has also recently demonstrated that VV therapy can be improved by utilizing the MM cell line KPMM2 as a cell-carrier. By expressing CXCR4, a critical regulator of myeloma cell homing to bone marrow, KPMM2 cells were found to be able to specifically deliver an attenuated VV to MM lesions in immunocompromised mice^104^. Although no clinical trials have been initiated to date, the recent activity utilizing different oncolytic VV in MM lends optimism for the future.

### Vesicular stomatitis virus (VSV)

VSV is an enveloped negative-stranded RNA virus that is a member of the *Rhabdoviridae* family. It replicates in the cytoplasm of infected cells and thus does not have the capacity to integrate in the host’s genome. It’s 11 kb genome encodes for only five proteins: nucleocapsid, polymerase proteins L and P, surface glycoprotein and a peripheral matrix protein. VSV infection can lead to a non-lethal but symptomatic infection in farm animals, but infection is essentially asymptomatic in humans^105, 106^. VSV cell entry is known to be largely mediated by the LDL receptor^107^. Notably, this virus is highly susceptible to the human innate immune response, so it selectively replicates in tumour cells that have deregulated immune response pathways, such as IFN^108, 109^.

An early pre-clinical study using an attenuated VSV with a NIS construct (VSVΔ51-NIS) for treatment of MM was completed by Goel and colleagues^110^. They showed that VSVΔ51-NIS had modest oncolytic potential against MM cell line and ex vivo patient samples. Furthermore, utilizing the 5TGM1 murine MM model, they demonstrated the potential for VSVΔ51-NIS in an in vivo system, where combination treatment with ^131^I radiotherapy led to reduction of tumour burden and improved survival^110^. More recently, it was postulated that adding an IFN-β construct to VSV would increase cancer cell susceptibility to infection and oncolysis while promoting viral clearance in non-cancerous tissues, as well as increase the potential for an adaptive anti-tumour immune response. Utilizing this model (VSV-IFNβ), Naik and colleagues^111^ showed that intravenous administration of this OV in the 5TGM1 model led to reduced disease burden and prolonged survival compared to controls—responses achieved with minimal toxicity. Further, one animal was completely cured of systemic disease of the study period. These pre-clinical results have led to the establishment of an early phase clinical trial (NCT03017820) of VSV-IFNβ-NIS in patients with hematologic malignancies, including relapsed or refractory MM. The trial is currently active, and open for recruitment.

## Optimization of OV in MM

The potential success of any OV is inextricably linked to its ability to induce a systemic anti-tumour immune response. As discussed, the general immunosuppressive environment that surrounds MM makes it difficult to achieve a sufficient immune response with an OV alone. Results from the first completed clinical trial utilizing OV in MM support this notion^93^, as do results from clinical trials in other tumour sites^112^. Thus, future successes of OV in MM will largely depend on the ability to use synergistic treatment approaches to facilitate a potent and robust anti-tumour immune response. Two such promising strategies include combining OV with ICI, as well as immunomodulatory agents such as lenalidomide.

### Immune checkpoint inhibitors (ICI)

Based on the understanding of the role of immune checkpoints in MM, a host of clinical trials evaluating the clinical utility of ICIs are currently ongoing^31^. However, one factor that limits the use of ICI as a cancer therapy in general is that most patients are non-responders^113, 114^, and this is no different in MM^115^. Thus, a rational approach would be to use ICIs as one part of a multi-mechanistic immunotherapeutic approach, of which OVs are a logical component.

Impetus for this combinatorial treatment approach comes in part from the ongoing phase I clinical trial utilizing RV in MM (NCT02514382) conducted by Kelly and colleagues^96^. During interim analysis, they found that ex-vivo treatment of patient tumour samples led to a significant increase in MM cell expression of PD-L1, as measured by flow cytometry and RT-PCR^96^. Subsequent in vivo experiments by this group using the 5TGM1-luc murine model have shown that treatment with both Reolysin and a PD-L1 inhibitor lead to a decrease in disease burden, as well as a significant increase in overall survival compared to either monotherapy alone^116^. Analysis of bone marrow specimens from mice in all evaluated experimental groups confirmed that Reolysin increases PD-L1 levels in a way that was directly linked to the enhanced efficacy of dual therapy^116^. Work from our lab has also found that RV treatment of both MM cell lines, and the Vk*MYC murine model leads to upregulation of PD-L1 on MM cells, and we are currently evaluating the treatment synergy using Reolysin and PD-1 blockade in this model.(Unpublished data) Although not yet evaluated in MM, the use of VSV-IFNβ-NIS in combination with PD-L1 blockade has shown promising in vivo results in a mouse model of acute myeloid leukemia. ^117^Dual treatment led to a significant survival benefit in treated mice, and corresponded with an increase in tumour infiltrating CD4^+^ and CD8^+^ T cells^117^. Thus, the theoretical clinical applicability of ICI in conjunction with OV for hematologic malignancies, including MM is certainly promising.

Despite initial evidence that would rationally support the use of ICI for the treatment of MM, recent developments in clinical trials have demonstrated reason for concern. Two phase III clinical trials studying pembrolizumab in conjunction with an immunomodulatory agent and dexamethasone (KEYNOTE-183 and KEYNOTE-185) have been halted by the FDA due to interim analyses demonstrating an increased risk of death in the pembrolizumab arm as compared to the control arm. It remains to be seen, however, how these findings will impact the clinical utility of ICI for MM moving forward. As such, the optimism generated by the pre-clinical efficacy of these agents for the treatment MM will be on hold until the reasons for the increased risk of death are elucidated.

### Immunomodulatory drugs (IMiDs)

The introduction of IMiDs like the thalidomide derivatives lenalidomide and pomalidomide into the clinical arena has represented a paradigm shift in the treatment of MM. IMiDs have contributed to the substantially improved outcomes seen in MM patients over the past decade and as such, their role as an anti-MM agent is well established^2^. The effects induced by IMiDs are pleiotropic but as their name suggests, they play an important role in modulating the inherently immunosuppressive environment of MM. Specifically, they have been shown to co-stimulate partially active T cells^118^, enhance NK cell proliferation^119^, inhibit proliferation and function of Tregs^120^, and downregulate the PD-L1/PD-1 pathway in MM^25, 121^. In addition to the immune effects, IMiDs are well understood to have roles in abrogating MM angiogenesis, altering adhesion between MM cells and the bone marrow environment, and mediating direct cell death through the induction of apoptosis^122^. Their established clinical role in MM and their immune-modulatory effects make them a logical candidate for treatment synergy with OV.

A pre-clinical study conducted by Parrish and colleagues^123^ using an oncolytic RV and lenalidomide highlights the potential of this treatment approach in MM. This group found that lenalidomide had augmented anti-tumour efficacy against *ex vivo* human MM cells and MM cell lines when combined with RV. Furthermore, dual treatment abrogated the cytoprotection of MM cells against lenalidomide that is typically offered by culture with bone marrow stromal cells^123^. These pre-clinical findings have been leveraged into an early phase clinical trial of Reolysin and IMiDs in MM, which is currently active but not yet recruiting. (NCT03015922) Interestingly, it has also been shown the PD-1/PD-L1 blockade in MM be enhanced with lenalidomide by further attenuating MDSC-mediated immune suppression and abrogating bone marrow stromal cell-induced MM growth^27^. Thus, another potential robust immunotherapeutic approach would be combining IMiDs, ICI and OV for the treatment of MM.

## Concluding remarks

OV represent a promising immunotherapeutic approach for the treatment of MM. Although there is insufficient data to currently support their use in the clinical arena as monotherapy, their ability to potentiate a systemic anti-tumour immune response make them a logical candidate to be synergistically combined with other immunotherapies such as ICI and IMiDs. As the current slate of clinical trials investigating the efficacy of oncolytic MV, RV and VSV in MM begin to unfold, time will tell if they have a permanent role as part of an immune-based treatment regimen for our patients. Finally, recent pre-clinical developments in MYXV and VV serve as motivation for continued investigation of OV for the treatment of MM.
